# The “Dogs’ Catching Mice” conjecture in Chinese phonogram processing

**DOI:** 10.1371/journal.pone.0324848

**Published:** 2025-06-06

**Authors:** Meng Jiang, Qi Luo, Xia Wang, Ya Tan

**Affiliations:** 1 College of Language Intelligence (College of General Education), Sichuan International Studies University, Chongqing, China; 2 Language & Brain Research Center, Sichuan International Studies University, Chongqing, China; 3 Chongqing Shapingba District International Joint Institute of Brain Computer Language Interface, Chongqing, China,; 4 School of English Studies, Sichuan International Studies University, Chongqing, China; Hong Kong Baptist University, HONG KONG

## Abstract

In Chinese phonogram processing studies, it is not strange that phonetic radicals contribute **phonologically** to phonograms’ phonological recognition. The present study, however, based on previous findings of phonetic radicals’ proneness to semantic activation, as well as free-standing phonetic radicals’ possession of triadic interconnections of orthography, phonology, and semantics at the lexical level, proposed that phonetic radicals may contribute **semantically** to the host phonograms’ phonological recognition. We label this speculation as the *“Dogs’ Catching Mice” Conjecture*. To examine this conjecture, three experiments were conducted. **Experiment 1** was designed to confirm whether phonetic radicals, when embedded in phonograms, can contribute semantically to their host phonograms’ phonological recognition. **Experiment 2** was intended to show that the embedded phonetic radicals employed in Experiment 1 were truly semantically activated. **Experiment 3,** on top of the first two experiments, was devoted to demonstrating that the semantically activated phonetic radicals, when used as independent characters, can truly contribute semantically to their phonological recognition. Results from the three experiments combine to confirm the conjecture. The implication drawn is that phonetic radicals may have forged two paths in contributing to the host phonograms’ phonological recognition: one is the regular *“Cats’ Catching Mice” Path*, the other is the novel *“Dogs’ Catching Mice” Path.*

## 1. Introduction

### 1.1 Properties of Chinese characters and phonetic radicals

The Chinese writing system exhibits a hierarchical structure with three levels: strokes, radicals, and characters. Strokes are the smallest units without semantic meanings that combine to form characters. Radicals, formed by strokes, serve as meaningful units and act as the basic building blocks for characters. Characters are functional logograms that can either consist of multiple radicals or be directly constructed by strokes. The distinction between radicals and characters could be blurred [1,2], as the same group of strokes can be considered as a “character” when standing alone but becomes a “radical” when combined with others. This gives rise to the distinction of simple characters and complex characters in Chinese. Simple characters, such as “口” (/kou3/, mouth), “门” (/men2/, door), and “贝” (/bei4/, shell), have straightforward and holistic structures that often bear resemblance to the objects they represent. They can also serve as radicals by which complex characters are constructed. Complex characters, making up the majority of Chinese characters, take on several structural patterns, such as top-bottom (e.g., “员”-/yuan2/-“member”), left-right (e.g., “呗”-/bei1/-“to chant”), and enclosing (e.g., “问”-/wen4/-“to ask”). Moreover, these complex characters can further combine with other components to form more intricate characters, such as “员” (member) enclosed in “圆” (circle).

Over 85% of Chinese complex characters are known as ideophonetic compound characters or phonograms [3], comprising two functionally different radicals, namely, phonetic radicals and semantic radicals [4–6]. Among the frequently-used radicals, there are approximately 800 phonetic radicals and 200 semantic radicals [7]. As their names suggest, phonetic radicals indicate the pronunciation of the characters, while semantic radicals provide semantic cues to the phonograms [4]. For instance, the phonogram “烤” (/kao3/, to bake) contains the semantic radical “火” (/huo3/, fire) locating at the left side, which denotes the instrumental characteristics of the whole character, and the right-side phonetic radical “考” (/kao3/, to test) gives hints to the whole character’s pronunciation “kao3”. Depending on the degree of similarity in pronunciation between the phonogram and its embedded phonetic radical, namely, ***phonetic regularity***, Chinese phonograms are classified into regular, semi-regular (or partially regular), and irregular ones [8]. ***Regular*** phonograms share the same pronunciations (same syllables and tones) with their phonetic radicals (e.g., phonogram “枫”/feng1/, maple; phonetic radical “风”/feng1/, wind). ***Semi-regular*** phonograms share the same rime but different tones (e.g., phonogram “妈”/ma1/, mother; phonetic radical “马”/ma3/, horse), or similar phonemes (e.g., phonogram “愧”/kui4/, ashamed; phonetic radical “鬼”/gui3/, ghost), with their phonetic radicals. ***Irregular*** phonograms possess distinct pronunciations with their embedded phonetic radicals (e.g., phonogram “猜”/cai1/, guess; phonetic radical “青”/qing1/, green) [5,9–11]. It is worth noting that only approximately 35−40% of phonograms are regular characters, while the remaining phonograms are semi-regular (30%) or irregular ones (30%−35%) [12].

### 1.2 Studies addressing phonetic radicals’ phonological contribution to Chinese phonograms’ phonological recognition

Existing studies have predominantly focused on examining the phonological activation of phonetic radicals and their contributions to the whole characters’ phonological recognition. Phonological representations of phonetic radicals are activated during Chinese character recognition, regardless of the phonetic regularity of the host characters [13–16]. For instance, in primed character naming experiments, irregular primes were found to facilitate the naming of the targets that were homophonic with the phonetic radicals embedded in the primes [17]. Also, prior studies have explored the temporal dynamics of phonological activation of phonetic radicals and their host phonograms, which demonstrated that the pronunciations of phonetic radicals were activated pre-lexically, preceding those of their host phonogram characters [18]. For example, Zhou et al. [19] explored the relative temporal order of phonological activation of phonetic radicals and their host characters by comparing the interference effects of radical-related and character-related primes. In their study, event-related potentials (ERPs) were recorded to capture the neural dynamics of this process. The N170 and P200 components, early ERP responses peaking around 170 ms and 200 ms respectively, are typically associated with early visual-orthographic analysis and phonological processing, with larger amplitudes reflecting greater processing demands. The N400 component, a later negative deflection peaking around 400 ms, is well known to index the difficulty of semantic integration, with larger amplitudes indicating increased semantic processing difficulty. The results revealed an early radical-related inhibitory effect, characterized by enhanced N170 and P200 amplitudes when the prime was related to the phonetic radical. This suggests that radical information engaged early visual and phonological processes, increasing the cognitive load during early stages of recognition. A much later character-related inhibitory effect was also observed in N400, indicating greater difficulty in integrating the phonological and semantic information at the whole-character level.

It has been no less firmly established that the phonological activation of phonetic radicals can significantly facilitate the recognition of the embedding characters. This conclusion builds on an extensive body of research utilizing phonological-oriented tasks) [18], such as homophone judgement task (or character sound-matching task) [20–22], phonological primed naming task [17,23], and pronunciation judgement task [24]. For instance, Chen and Allport [20] asked participants to judge whether two characters were homophones, and they found that participants responded faster when the two target characters, which had different pronunciations, contained different phonetic radicals, as compared to when they contained the same phonetic radicals. In another study by Zhang et al. [24], characters and their embedded phonetic radicals’ pronunciation repetition were manipulated during the continuous presentation of four Chinese characters. The participants were tasked with determining whether the 4th character’s pronunciation began with the consonant “g”. The results showed greater neural adaptation in the left hemisphere’s N170 when the four continuously presented characters shared the same phonetic radical, as compared to when they possessed different ones. The left N170 component, an early negative ERP deflection peaking around 170 ms, is sensitive to visual-orthographic and phonological processing. Specifically, larger N170 amplitudes are typically associated with increased phonological processing demands, such as when integrating complex or unfamiliar phonological information, whereas reduced amplitudes indicate more efficient processing due to facilitated access to phonological representations. Thus, the observed reduction in N170 amplitude for shared phonetic radicals suggests that these radicals reduce the difficulty of phonological processing, thereby facilitating the phonological activation of the host characters [25]. Likewise, Hung et al. [22] utilized the MEG technique to investigate the role of phonetic radicals in Chinese characters’ recognition. In their first experiment, participants engaged in a homophone judgment task. They were asked to determine whether prime and target characters were homophones. Both reaction times and MEG results demonstrated a facilitatory effect when the prime and target characters shared the same phonetic radicals, indicating phonetic radicals’ contribution to the whole characters’ phonological recognition.

It has also been found that *phonetic regularity* bears on the speed of character naming. The explanation is that *phonetic regularity* could affect the reliability of the mapping between the pronunciations of phonograms and their phonetic radicals [2,5]. For instance, Yum et al. [26] manipulated the regularity of Chinese characters in delayed naming tasks and found a significant regularity effect in the N170 component, with regular characters inducing larger N170 amplitudes than irregular ones. This effect, emerging within 200 ms post-stimulus onset, reflects the orthographic analysis of phonograms into radicals and their phonological mapping. Larger N170 amplitudes for regular characters suggest efficient processing due to the absence of conflict between the phonological forms of the phonogram and its phonetic radical. In contrast, smaller N170 amplitudes for irregular characters indicate conflict, requiring additional cognitive resources for resolution. These findings highlight the role of phonetic regularity in modulating early phonological processing during character naming. Similar results were also found by Hue [27] and Lee et al. [28]. To summarize, the phonological activation of phonetic radicals can facilitate the phonological recognition of the host characters, and this facilitatory effect can be modulated by the phonetic regularity of the host characters.

### 1.3 The *“Dogs’ Catching Mice” conjecture*

It is not strange that phonetic radicals contribute phonologically to phonograms’ phonological recognition, just in the same way that semantic radicals contribute semantically to phonograms’ semantic recognition [29–31]. It describes little more than the common sight of a cat’s catching a mouse. What deserves special attention is whether phonetic radicals can contribute semantically to phonograms’ phonological recognition. This assumption can be conveniently labeled the “Dogs, in place of cats, catch mice” conjecture, or the *“Dogs’ Catching Mice” Conjecture* for short.

Two lines of research lend support to this enticing speculation. The first line of research captures phonetic radicals’ proneness to semantic activation. Quite a few studies have investigated the semantic activation of phonetic radicals in Chinese character recognition, consistently demonstrating that phonetic radicals can indeed activate semantic information. These studies have utilized a range of tasks, including semantic-unrelated tasks such as the primed character naming task [2], character recognition task [5], lexical decision task ( [32], Experiment 1), the Stroop task [1], and only one semantic-related task, i.e., semantic categorization task ( [32], Experiment 2). Specifically, Zhou and Marslen-Wilson’s pioneering research [14] yielded compelling evidence that the meaning of phonetic radicals were indeed activated in the processes of characters’ recognition. They revealed a significant effect whereby participants responded more quickly when the target characters were semantically related with the phonetic radicals embedded in the prime characters, even when the prime and target characters bore no semantic connection at the whole-character level. Similarly, Lee et al. [5] utilized the strength of N400 as an indicator and observed a semantic priming effect of N400 when the phonetic radicals of the regular and irregular prime characters were semantically related to the targets, which indicated the semantic activation of the phonetic radicals during phonograms’ recognition. Later, Tsang et al. [32] endeavoured to replicate the findings of [14] and [5] and revealed that during the character recognition process, phonetic radicals can activate both their own semantic and phonological knowledge just like their host characters. In the same year, Yeh, Chou and Ho [1] employed a Stroop task wherein participants were instructed to judge the colour of the target characters. The character stimuli incorporated phonetic radicals that were either exact colour names (e.g., “青”, meaning “green”) or colour-related objects (e.g., “血”, meaning “blood”). Stroop effects were found when the colours manifested by phonetic radicals interfered with the colours of target characters (e.g., target character “猜” in red with its phonetic radical “青”; or target character “恤” in green with its phonetic radical “血”). These findings suggested that the semantic meanings of phonetic radicals were activated during the recognition process of the entire characters. The significance of these observations lies in the reasoning that once the semantic information of the embedded phonetic radicals is activated, it may have to seek a role to play in the whole character’s recognition.

Another line of research highlights the triadic interconnections and thus mutual access of Chinese independent character’s orthographic, phonological, and semantic representations. Chinese is a morpho-syllabic language wherein each character represents a self-contained unit. According to the Lexical Constituency Model [33], a character’s representation consists of three interlocking constituents: orthographic, phonological, and semantic information. Successful lexical retrieval or character identification requires the complete accessing of all the three constituents [13]. Similarly, the Connectionist Theory suggests that learning and comprehending Chinese characters necessitates the integration of these three types of information: visual form, phonology, and semantics [34]. The shared point of the model and the theory is that the three types of lexical information of Chinese characters are mutually accessible. The activation of one may lead to the activation of the other two. For example, the phonological representation, once activated, may well lead to the activation of the semantic as well as the orthographic representations.

In Chinese, presumably over 85% of phonetic radicals can serve as stand-alone characters [35,36], indicating that they possess their own phonological, orthographic, and semantic information and that the three types of information are highly integrated [1,37]. When they serve as sub-lexical units in phonograms, what applies to their lexical representations may well apply to their sub-lexical representations. In the case of a host phonogram, even when the phonetic radical is overwhelmed with the function of providing phonological clues to the whole character, its sub-lexical representations might indeed inherit the interconnection properties of its previous lexical representations. That is, a free-standing phonetic radical, when embedded in a host phonogram, may still embody its previous lexical phonological, orthographic and semantic interconnections. The radical’s sublexical-level phonological activation is highly likely to beget both the sub-lexical-level semantic and orthographic activation.

**Fig 1:**
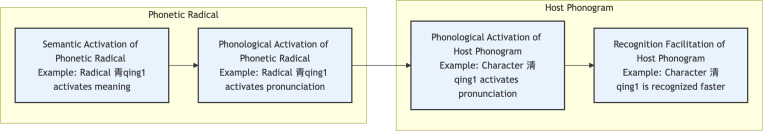
Time Course of the “*Dogs’ Catching Mice” Path* in Phonetic Radical Activation during Chinese Phonogram Recognition.

All things taken together, it is highly probable that a phonetic radical could contribute semantically to its host phonogram’s phonological recognition. The unconventional path blazed thereby may be: the phonetic radical’s semantic activation → the phonetic radical’s phonological activation → the host phonogram’s phonological activation → the host phonogram’s phonological recognition facilitated. To elaborate a little bit, the phonetic radical first activated its sub-lexical semantic information. This activation may spread to the radical’s sub-lexical phonological information, which warms up the whole phonogram’s phonological information retrieval, ultimately resulting in an accelerated phonological recognition of the phonogram. As such, this path can be conveniently called the *“Dogs’ Catching Mice” Path*, in relation to the *“Cats’ Catching Mice” Path* wherein the phonetic radical contributes phonological hints directly to its host phonogram’s phonological recognition. This sequence is supported by the Cascaded Activation phenomenon (e.g., [14]), which posits that semantic activation precedes phonological activation in logographic systems like Chinese. Empirical evidence from priming studies (e.g., [5]) further supports this timeline. To enhance clarity, we have included a diagram (Fig 1) illustrating the proposed time course of activation and its interaction with whole-character representation. To recapitulate, we posit that phonetic radicals may have blazed two paths in contributing to their phonograms’ phonological recognition: the commonly-accepted, more direct *“Cats’ Catching Mice” Path*, and the largely-ignored somewhat indirect *“Dogs’ Catching Mice” Path*. Great efforts are taken to address the former, but hardly any heed is paid to the latter.

### 1.4 The present study

The present study aims to examine the above *“Dogs’ Catching Mice” Conjecture* by first investigating whether the **semantic** activation of phonetic radicals can influence the **phonological** recognition of their host phonograms, yielding what can be called the “*Dogs’ Catching Mice*” effect. Then this study delved into the possible machinery working behind. Toward this purpose, three experiments were conducted. **Experiment 1** was designed to confirm whether phonetic radicals, when embedded in phonograms, can contribute semantically to their host phonogram’s phonological recognition. The variable *phonetic regularity* (i.e., regular, semi-regular, irregular) was manipulated. It is predicted that regularity would moderate the *“Dogs’ Catching Mice” effect*, with the regular phonograms exhibiting the largest size of *“Dogs’ Catching Mice” effect*, the semi-regular phonograms the smaller, and the irregular ones the smallest (or even no effect at all).

**Experiment 2,** differing from Experiment 1 only in the task employed, used a primed part-of-speech judgment task to explore whether the embedded phonetic radicals were truly semantically activated. While previous studies have provided evidence for semantic activation, our study addresses several limitations in the literature. First, unlike prior research that embedded phonetic radicals in prime characters, we embedded them in target characters. Second, we employed a part-of-speech judgment task, which avoids the memorization demands of semantic categorization tasks and the narrow focus of Stroop tasks (e.g., [1], which only examined color-related radicals), ensuring that observed effects are solely attributable to semantic activation. Third, our study included semi-regular characters, expanding the scope of prior research, which primarily focused on regular and irregular phonograms. These methodological refinements allow us to provide more robust and generalizable evidence for the semantic activation of phonetic radicals. We hypothesized that the semantic information associated with phonetic radicals can be activated and, in turn, interfere with the semantic processing of their semantically unrelated host characters. **Experiment 3,** on top of the first two experiments, was designed to demonstrate that the semantically activated phonetic radicals, when used as independent characters, can truly contribute semantically to their phonological recognition, documenting *“Dogs’ Catching Mice” effects* at the lexical level. While the Connectionist Theory [34] posits a general relationship between phonological and semantic representations, empirical validation is essential to confirm its applicability to specific linguistic phenomena, particularly in the context of semi-regular phonograms, which have not been systematically investigated. By testing whether the semantic activation of phonetic radicals influences phonological recognition, this experiment extends prior research and provides robust evidence for the robustness of this relationship across different character types. This not only strengthens the theoretical framework but also offers practical insights into the cognitive mechanisms underlying Chinese character processing.

## 2. Experiment 1

### 2.1 Method

#### 2.1.1 Participants.

Thirty participants were recruited in the experiment who were native Mandarin speakers living in mainland China (mean age = 26.4 years, *SD* = 4.47). All of them were students from Sichuan International Studies University (SISU). They were right-handed, and possessed normal or corrected-to-normal vision. The participants for this study were recruited from September 15, 2023 to November 15, 2023. They were required to provide written informed consents prior to the formal experiment, and would receive compensation after completing the experiment.

#### 2.1.2 Materials and design.

Experiment 1 adopted a *2 (prime type: semantically related vs. semantically unrelated characters) × 3 (target type: regular vs. irregular vs. semi-regular characters)* design. Concerning the two prime types, each of them contained 30 Chinese characters (see Table 1 for sample materials and their properties): (1) **The semantically related (SR)** prime characters (e.g., “时-/shi2/-time”) were semantically related to the target characters (e.g., “裱-/biao3/-paste up”) solely at the phonetic-radical (e.g., “表-/biao3/-watch”) but not at the whole-character level; (2) **The semantically unrelated (SU)** prime characters (e.g., “柜-/gui4/-cupboard”) were semantically, orthographically, or phonologically unrelated to the targets (e.g., “裱-/biao3/-paste up”) either at the radical level or at the whole-character level. The two types of primes were matched in the character’s structure, number of strokes (*t*(29) = −1.030, *p* = .312), part of speech, and mean frequency (*t*(29) =.778, *p* = .443).

**Table 1:** Sample materials and their properties in Experiment 1.

Primes		Targets		Free-standing Phonetic Radicals Embedded in the Targets
Semantically Related (SR)	Semantically Unrelated (SU)			
时 /shi2/-time	柜 /gui4/-cupboard	裱 /biao3/-to paste up	Regular Phonogram	表 /biao3/-watch
爬 /pa2/-climb	选 /xuan3/-to select	橙 /cheng2/-orange	Semi-regular Phonogram	登 /deng1/-to step on
脚 /jiao3/-foot	辫 /bian4/-braid	捉 /zhuo1/-to catch	Irregular Phonogram	足 /zu2/-foot

With regard to the target types, 30 Chinese phonograms were divided into three categories according to their **phonetic regularity** (10 phonograms for each condition): (1) **Regular phonograms** (e.g., “裱-/biao3/-paste up”) had the same pronunciations with their embedded phonetic radicals (e.g., “表-/biao3/-watch”); (2) **Semi-regular phonograms** (e.g., “橙-/cheng2/-orange”) and their phonetic radicals (“登-/deng1/-step on”) shared similar phonemes (see Table 1 for sample materials); (3) **Irregular phonograms** (e.g., “捉-/zhuo1/-catch”) were phonologically distinct from their phonetic radicals (e.g., “足-/zu2/-foot”). All of these phonograms were semantically unrelated to their phonetic radicals (e.g., the meaning of the phonogram “橙” [meaning “orange”] and its phonetic radical “登” [meaning “climb”] differed significantly with that of the host character). The full set of materials is available in S1 Table.

The semantic relatedness between the prime and the target characters (as well as the phonetic radicals embedded in the targets) was assessed by a group of 25 native Chinese speakers who did not participate in the experiment. They completed a 7-point Likert scale ranging from 1 (not related at all) to 7 (extremely related). The rating results suggested a high level of semantic relatedness (6.002) between the semantically related (SR) primes and the embedded phonetic radicals of the targets, and a low level of semantic relatedness (1.704) between the the semantically unrelated (SU) primes and the embedded phonetic radicals of the targets. The semantic relatedness, either between the SR prime and the target characters (1.653) or between the SU primes and the target characters (1.353), was also low.

As the target characters, which were used for a readability judgement task, were all authentic Chinese characters, 30 pseudo-characters were constructed as fillers by removing strokes from or adding strokes to real Chinese characters. Altogether, thirty pairs of primes (SR and SU primes) were matched with 30 real targets and 30 pseudo-character targets, resulting in a total of 120 prime-target pairs which were fully utilized across trials.

#### 2.1.3 Procedure.

The stimuli were presented on a 15-inch CRT monitor, utilizing the E-Prime 2.0 (Psychology Software Tools, Inc.) software. The characters were presented in the font Song in black with a white background color. Participants underwent the testing in an electrically and magnetically shielded room, being comfortably seated at approximately 60 cm from the computer. With this viewing distance, each character occupied a visual angle of 1° in both width and height when encircled.

Participants first pressed Q to enter a 6-trial practice session and received feedback to familiarize with the procedure. Afterward, they could press “P” and proceeded to the formal experiment, which comprised 120 randomized trials. Concerning the formal experiment, each trial began with an eye fixation signal in the form of a cross-shaped “+” for 300ms, followed by a prime character appearing for 500ms. Then the prime was replaced by a target character (either a real or a pseudo-character), which remained visible until the participants responded. This was followed by a 600ms blank screen interval. During the experiment, breaks between test sessions were permitted.

Participants were instructed to ascertain whether the target character was readable or not as quickly as possible by pressing the key “J” or “F”. The key configuration was counterbalanced across participants so that for half of them, the “F” key indicated a “YES” response and “J” key representing “NO”, while the other half followed the reverse configuration. Accuracy and reaction times were recorded. On average, each participant took approximately 10 minutes to complete the entire experiment. This experiment was conducted in the Key Laboratory of Foreign Language Learning and Cognitive Neuroscience in SISU.

### 2.2 Results

Data of 30 participants were included in the statistical analyses. The mean accuracy was 96.25%. The trials with incorrect responses or reaction time exceeding ±2 *SD* (1.77%) from the mean reaction time (RT) of each condition were excluded from further analyses. The results were displayed in Table 2.

**Table 2:** Mean RT (ms) and *SD* for target judgement in Experiment 1.

Semantic Relatedness	Phonetic Regularity		
	Regular	Semi-regular	Irregular
**Semantically Related (SR)**	549.424 (76.214)	580.227 (86.762)	591.266 (99.831)
**Semantically Unrelated (SU)**	601.637 (115.041)	592.825 (89.986)	592.753 (89.331)

A two-way repeated-measures ANOVA was performed on the RTs. Results showed that the main effect of the **Semantic Relatedness** was significant (*F*(1, 29) =6.105, *p* = .020, *η*^2^ = .174). The main effect of the **Phonetic Regularity** was not significant (*F*(1.384, 40.136) =1.099, *p* = .322, *η*^2^ = .037). The interaction between the **Semantic Relatedness** and the **Phonetic Regularity** was significant (*F*(2, 58)=4.846, *p* = .011, *η*^2^ = .143).

The simple effect analysis showed that the RTs to *Regular Phonogram* targets preceded by semantic-related primes (549.424ms) were significantly faster than those preceded by semantically unrelated primes (601.637ms) (*F*(1, 29) =11.757, *p* = .002, *η*^2^ = .288). The RTs to Semi-regular Phonogram targets preceded by semantically-related primes (580.227ms) were marginally faster than those preceded by semantically unrelated primes (592.825ms) (*F*(1, 29) =.009, *p* = .085, *η*^2^ = .099). The RTs to Irregular Phonogram targets preceded by semantic-related primes (591.266ms) did not significantly differ from those preceded by semantically unrelated primes (592.753ms) (*F*(1, 29) =3.179, *p* = .926, *η*^2^ = .000). These results indicate that priming effects, or *“Dogs’ Catching Mice” effects*, occurred to both Regular and Semi-regular Phonogram targets, but not to Irregular Phonogram targets.

Further statistical analyses were run on the RT differences (i.e., RT semantically-unrelated primes – RT semantically-related primes) for the three types of targets. Significant differences were found between Regular and Semi-regular Phonograms (*F*(2, 28) =4.787, *p* = .024, *η*^2^ = .255). Marginal significant differences were found between Regular and Irregular Phonograms (*F*(2, 28) =4.787, *p* = .052, *η*^2^ = .255), while no significant differences were found between Semi-regular and Irregular Phonograms (*F*(2, 28) =4.787, *p* = 1.000, *η*^2^ = .255). These results suggest that the regular targets had produced the largest size of *“Dogs’ Catching Mice” effects*, with the semi-regular targets the smaller, and the irregular targets no effects at all.

### 2.3 Discussion

The findings of Experiment 1 indeed captured the *“Dogs’ Catching Mice” effects.* Phonetic radicals embedded in the regular as well as semi-regular target phonograms had ***semantically*** facilitated the host phonograms’ ***phonological*** recognition. Based on the experimental design, there was no obvious phonological or semantic relationship between the primes and targets. The semantic relationship between the prime and the phonetic radical embedded in the target character should be held responsible for the facilitatory effects. In other words, the phonetic radicals had contributed semantically to the phonological recognition of their host phonograms. This finding was rather novel in that previous studies only revealed that the semantic information of phonetic radicals could be truly activated in the process of phonograms’ recognition [2,5,32]. No known studies, however, had investigated what the activated semantic information of phonetic radicals could do, or simply what role it could necessarily play. The present study, noticeably, pushed a step forward in this respect by demonstrating that the semantic activation of phonetic radicals could conduce to the phonological recognition of their embedding phonograms. That is, it could find a role in assisting with the host character’s phonological retrieval.

As mentioned previously, phonetical radicals, by definition, serve to provide phonological cues or clues for the host phonogram’s phonology [4], thereby capable of assisting with the phonogram’s pronunciation by phonologically activating themselves at the sub-lexical level [16,18]. However, our findings suggest that phonetic radicals can also contribute to phonological processing through an alternative pathway: semantic self-activation at the sub-lexical level. This raises the question: How can phonetic radicals, which are traditionally associated with phonological activation, also influence phonological processing through semantic activation? This amounts to the phonetic radical’s semantic representation exceeding its originally-defined duties (though there seem to be no stipulated duties for it) and meddling in its phonological representation’s affairs, analogous to the dog’s meddling in the cat’s affairs (That’s why we call this phenomenon *“Dogs’ Catching Mice” effects*). In reference to the triadic mental interconnections of phonology, orthography, and semantics of an independent character, as cited copiously in the literature [13,33,34], a plausible reasoning may be that what happens to an independent character at the lexical level may have as well happened to the same character embedded in a phonogram as a sub-lexical radical [32]. The hidden path may be characterized this way: The phonetic radical’s sub-lexical-level semantic activation may have led to its sub-lexical-level phonological activation, which in turn may have primed the host character’s lexical-level phonological retrieval, which finally resulted in a sped-up phonological recognition of the host character. This path may have stood as an extraordinary or unconventional machinery that underlies phonograms’ regular processing but has been more than often ignored. This posited path or machinery, in combination with its outward manifestations, namely, the *“Dogs’ Catching Mice” effects,* constitutes what we refer to as *the “Dogs’ Catching Mice” Conjecture.*

Meanwhile, the pattern of results of Experiment 1 also aligned with the predictions of the graded *“Dogs’ Catching Mice” effects*. It was expected that the magnitude of *the “Dogs’ Catching Mice” effects* might be contingent upon the *phonetic regularity* of the phonograms wherein the phonetic radicals were embedded. This was truly confirmed. On the one hand, the priming effects, or more precisely, *the “Dogs’ Catching Mice” effects,* were found only in regular and semi-regular phonograms but not in irregular counterparts. Additionally, a larger size of *the “Dogs’ Catching Mice” effects* were found in regular phonograms as compared to semi-regular and irregular counterparts. One plausible explanation may be that in regular phonograms, the semantic activation of the embedded phonetic radicals led to their own phonological activation, which offered much stronger or more valid phonological pre-activation [5] to the host phonograms, ultimately resulting in a rapid and efficient activation of phonological information for the phonograms. In the semi-regular phonograms, the phonetic radicals’ phonological activation triggered by their semantic activation, could only offer a much weaker or less valid phonological pre-activation [5] to the host phonograms, ultimately resulting in a less rapid and less efficient activation of phonological information for the phonograms. In the case of irregular phonograms, by contrast, the phonetic radicals’ phonological activation arising from their semantic activation could almost have nothing to do to help with the phonogram’s phonological retrieval. The supporting fact is that, regular phonograms, relative to semi-regular or irregular phonograms, by definition, have much more to share with their embedded phonetic radicals [38,39] and thus have greater potency in phonologically pre-activating each other.

The semantic relationship between the prime and the phonetic radical embedded in the target character is responsible for the facilitatory effects observed in the phonetic regular and semi-regular conditions. In other words, the phonetic radicals contributed semantically to the phonological recognition of their host phonograms. However, the absence of inhibitory effects in the phonetic-irregular condition requires careful consideration. First, the phonological irregularity of the host characters inherently disrupts the connection between the phonetic radical’s phonological representation and the host character’s pronunciation. In regular and semi-regular characters, the phonetic radical’s phonological information aligns with or partially aligns with the host character’s pronunciation, creating a pathway for semantic activation to facilitate phonological processing. In contrast, irregular characters lack such phonological commonality, effectively severing this pathway. As a result, even if the semantic information of the phonetic radical is pre-activated by the prime, the absence of a shared phonological link prevents the transfer of this activation to the host character’s phonological representation. This explains why no inhibitory effects were observed in the irregular condition: the semantic activation of the phonetic radical simply does not propagate to the phonological level in the absence of a valid phonological pathway. Second, while collocational associations (e.g., “时” and “表” in “时间表”) might contribute to facilitation in some cases, such effects are limited to a small subset of prime-target pairs and cannot account for the consistent facilitatory effects observed across regular and semi-regular conditions. Additionally, the facilitatory effects in semi-regular characters, where the phonological overlap between the phonetic radical and the host character is only partial, further support the role of phonetic radicals in mediating these effects rather than collocational associations. This suggests that the semantic activation of phonetic radicals can still facilitate phonological processing even when the phonological link is weak. Third, in irregular characters, the semantic activation of phonetic radicals may simultaneously generate two opposing effects: (1) a facilitatory effect, where semantic activation indirectly enhances phonological processing through semantic networks, and (2) an inhibitory effect, where the phonological activation of the phonetic radical conflicts with the host character’s phonological representation. These opposing effects may cancel each other out, resulting in no net facilitation or inhibition in the irregular condition. This interpretation aligns with the proposed *“Dogs’ Catching Mice” Path*, where the phonological regularity of the host character determines the transfer of sub-lexical semantic activation to lexical-level phonological processing. Finally, one reviewer of the paper raises an important alternative explanation: the facilitatory effects in the regular condition might be mediated by phonological neighborhoods rather than the phonetic radicals themselves. For example, primes (e.g., 时, “time”) could semantically activate related words (e.g., 表, “watch”) that share the same pronunciation as the target, indirectly facilitating the phonological activation of the target words. While this is a plausible interpretation, our design carefully controlled for such confounds by ensuring that primes and targets did not share direct phonological or semantic relationships outside the embedded phonetic radical. Furthermore, the consistent pattern of results across experiments, particularly the moderation effect of phonetic regularity, strongly supports the role of phonetic radicals in mediating the observed effects. Nevertheless, we acknowledge that phonological neighborhoods could play a complementary role, and future studies should explicitly investigate their contribution.

Experiment 1 has well served the purpose of confirming the phonetic radicals’ semantic contribution to the phonological recognition of their host phonograms. It has also verified the moderation role that the variable *phonetic regularity* could play in this process. However, it offered little in testifying to the path or machinery that gave rise to the so-called *“Dogs’ Catching Mice” effects.* Toward this end, two more experiments were designed. The following experiment, namely, Experiment 2, was intended to show that the embedded phonetic radicals, as recruited in Experiment 1, have undergone true semantic activations, adding consolidation to the *“Dogs’ Catching Mice”* path conjecture.

## 3. Experiment 2

### 3.1 Method

Experiment 2 was identical to Experiment 1 in terms of participants, materials, and design, except that a primed part-of-speech judgment task was adopted instead of the primed phonological readability task. In the part-of-speech judgment task, participants were required to determine the grammatical category (e.g., noun, verb, adjective) of a target word that was preceded by a prime word. This task is more semantically focused, as it requires participants to access and evaluate the meaning and syntactic role of the target word within a linguistic context. Unlike the phonological readability task, which focused on phonological activation and its influence on word recognition, the part-of-speech judgment task emphasizes semantic and syntactic processing, addressing questions about semantic and syntactic priming, the level of linguistic representation affected by priming, and the generalizability of priming effects to higher-level linguistic processing involving meaning and grammar.Experiments 2 and 1 were conducted one week apart to minimize potential carryover effects and prevent participants from memorizing the stimulus materials.

During the procedure, participants were seated approximately 50 cm from a computer screen in the university’s Key Laboratory. Each trial began with a 300 ms fixation cross, followed by a prime character displayed for 500 ms. The prime was then replaced by a target character, which remained on the screen until a response was made. Participants were instructed to determine whether the target verb was a noun or not as quickly as possible by pressing the “F” or “J” keys. The key-to-response mapping was counterbalanced across participants, with half using “F” for “noun” and “J” for “verb”, and the other half using the reverse configuration. Both accuracy and reaction times were recorded. Response accuracy and response time were recorded for each trial.

### 3.2 Results

Data of 30 participants were included in the statistical analysis. The mean accuracy was 91.17%. The trials with incorrect responses or reaction time exceeding ±2 *SD* (1.94%) from the mean RT of each condition were excluded from further analyses. Table 3 shows the mean reaction times for each condition.

**Table 3:** Mean RT (ms) and *SD* for target judgement in Experiment 2.

Semantic Relatedness	Phonetic Regularity		
	Regular	Semi-regular	Irregular
**Semantically Related**	746.510 (183.481)	812.293 (202.295)	750.113 (159.468)
**Semantically Unrelated**	706.100 (113.340)	764.483 (152.903)	721.073 (114.530)

A two-way repeated-measures ANOVA was performed on the RTs. Results showed that the main effect of the **Semantic Relatedness** was significant (*F*(1, 29) =7.867, *p* = .009, *η*^2^ = .213). The main effect of the **Phonetic Regularity** was significant (*F*(2, 58) =.10.681, *p* = .000, *η*^2^ = .269). The interaction between the **Semantic Relatedness** and the **Phonetic Regularity** was not significant, *F*(2, 58)=.341, *p* = .713, *η*^2^ = .012. The results showed inhibitory effects for all the phonogram targets, irrespective of the targets’ phonetic regularity, which indicates that the embedded phonetic radicals were truly semantically activated.

### 3.3 Discussion

A host \of studies, which utilized a wide range of tasks like primed character naming task [2], character recognition task [5], lexical decision task ( [32], Experiment 1), Stroop task [1], and semantic categorisation task ( [32], Experiment 2), have shown that phonetic radicals could be semantically activated at the sub-lexical level. But it remains uncertain whether the meaning of the phonetic radicals as embedded in the target phonograms and subjected to the phonological recognition task in Experiment 1, was activated or not. The observed inhibitory semantic priming effects across all three types of phonogram targets in Experiment 2, be it regular, semi-regular or irregular, suggested that the semantic information of the embedded phonetic radicals was truly activated. This pattern of results is consistent with findings in [14] and [32], suggesting that the phonetic regularity of the characters did not affect the semantic activation of the embedded phonetic radicals.

The phonetic radical, as its name suggests, chiefly assumes the role of providing phonological clues to the pronunciation of the host phonogram, in contrast to the semantic radical which is held responsible for providing semantic cues to the host phonogram. In most if not all cases, the phonetic radical bears nothing semantic in common with the host phonogram’ meaning [5], just in the same way the semantic radical hardly bears anything phonological in common with the host phonogram’s phonology [23] Should the meaning of the phonetic radical be activated, extra efforts would be needed to get it suppressed. This suppression would cause an interference or a delay in semantic-oriented processing of the phonogram. The present experiment, which employed the part-of-speech judgment task, fell exactly within the category of semantic-oriented character processing. In other words, the inhibitory effects observed in Experiment 2 should be ascribed to the semantic activation of the embedded phonetic radicals. Given that Experiment 2 and Experiment 1 differed only in the task employed (part-of-speech judgment task vs. readability judgment task), the findings of Experiment 2 would help to corroborate the belief that the embedded phonetic radicals in Experiment 1 had been semantically activated. This observation goes in alignment with the Model of Character Processing [32], which suggests that the meanings of phonetic radicals can be activated, even if they do not directly contribute to the meanings of the entire characters.

One possible reason why phonetic regularity modulates the effects of phonetic radicals in Experiment 1, but not Experiment 2, may be related to the specific nature of the tasks. Experiment 1 used a readability judgment task, which could have made the processing of phonetic information more crucial, leading to a greater reliance on phonetic regularity. In contrast, the part-of-speech judgment task in Experiment 2 may have placed less emphasis on phonetic regularity, thereby reducing its modulation effects.

By Experiments 1 and 2, so far, we have confirmed that phonetic radicals can indeed contribute **semantically** (in contrast to **phonologically)** to their host phonogram’s phonological recognition, taking the form of *“Dogs’ Catching Mice” effects*. The posited machinery behind was the transfer, from the lexical level to the sub-lexical level, of the embedded phonetic radicals’ triadic interconnections of phonological, orthographical and semantic representations in their characterhood. According to [33], an independent character’s mental representation consisted of three interlocking constituents: orthographic, phonological, and semantic information. The activation of one was well in position to activate any of the other two [13]. An abundance of studies has addressed the mutual accessibility of the three types of lexical representations [34]. What is unclear is whether or not the pre-activation of one type of information will prime otherwise-oriented processing of the whole character. Put differently, should the phonetic radicals, when used as independent characters, exhibit similar *“Dogs’ Catching Mice” effects*? Experiment 3 was designed precisely to address this inquiry.

## 4. Experiment 3

### 4.1 Method

#### 4.1.1 Participants.

The participants in Experiment 3 were identical to those used in Experiment 1 and 2. Expriment 3 was conducted one week after Experiment 2 to minimize potential carryover effects and prevent participants from memorizing the stimulus materials. The order of three experiments was also counterbalanced across participants to ensure any potential practice effects.

#### 4.1.2 Materials and design.

Experiment 3 adopted a single factor *(prime type: semantically related vs. semantically unrelated)* two-level design. Prime characters and their properties were the same as those in Experiment 1. As for the target characters, we selected the embedded **phonetic radicals** of the target characters in Experiment 1 and utilized them as independent target **characters** in this experiment. For instance, we extracted the phonetic radical “表” (/biao3/, “watch”) of the target character “裱” (/biao3/, “paste up”) from Experiment 1 and used “表” as the target character in this experiment. Each phonetic radical in Experiment 1 was used only once to construct the material set in this experiment.

Identical to the prime characters in Experiment 1, there were 30 phonograms for each priming type (see Table 4 for sample materials and their properties): (1) **the semantically related** prime characters (e.g., “时-/shi2/-time”) and the target characters (e.g., “表-/biao3/-watch”) exhibited semantic relevance at the whole character level but steered clear of being commonly-used collocations; (2) **the semantically unrelated** prime characters (e.g., “柜-/gui4/-cupboard”) were not semantically, orthographically, or phonologically related to the targets (e.g., “表-/biao3/-watch”) either at the whole character level or at the radical level. The full set of materials is available in S2 Table.

**Table 4:** Sample materials and their properties in Experiment 3.

Primes		Targets
Semantically Related (SR)	Semantically Unrelated (SU)	
时-/shi2/-time	柜-/gui4/-cupboard	表-/biao3/-watch
爬 /pa2/-to climb	选 /xuan3/-to select	登 /deng1/-to step on
脚 /jiao3/-foot	辫 /bian4/-braid	足 /zu2/-foot

As the target characters, which were used for a readability judgement task, were all authentic Chinese characters, 30 pseudo-characters were created by either adding strokes to or removing strokes from genuine Chinese characters. Thirty pairs of primes were matched with 30 real targets and 30 pseudo-character targets. In total, 120 prime-target pairs were used for two types of priming condition (i.e., 60 pairs for SR condition and 60 for SU conditions).

#### 4.1.3 Procedure.

The experimental task as well as the procedure of Experiment 3 was the same as that of Experiment 1. Experiment 3 stood one week apart from Experiment 2.

### 4.2 Results

Data of 30 participants were included in the statistical analysis. The mean accuracy for the primed phonological readability task was 95.88%. The trials with incorrect responses or reaction time exceeding ±2 *SD* (1.67%) from the mean RT of each condition were excluded from further analyses. Table 5 displays the mean reaction times for the remaining data.

**Table 5:** Mean RT (ms) and *SD* for target judgement in Experiment 3.

Semantic Relatedness	Target
**Semantically Related (SR)**	595.733 (93.549)
**Semantically Unrelated (SU)**	632.633 (95.331)

A paired samples *t*-test was employed to measure the RTs. The mean RTs to targets preceded by semantic-related primes (595.733ms) were significantly faster than those corresponding semantically-unrelated primes (632.633ms) (*t*(29) = −2.618, *p* = .014, Cohen’s d = 0.478). There was a significant priming effect. The results indicated that the character target’s semantic activation could contribute to its own phonological recognition in a facilitatory manner.

### 4.3 Discussion

The results of Experiment 3 shed light on the contribution of a character’s semantic activation to its phonological recognition, documenting the character-level or lexical-level *“Dogs’ Catching Mice” effect*. Based on the experimental design, when free-standing phonetic radicals were extracted from their host phonograms and functioned independently as characters, their semantic activation could assist with their phonological retrieval and thereby speed up the phonological recognition of themselves. Previous researchers have reported that during the visual processing of Chinese characters, the semantic activation of a character would instantaneously and automatically transfer to its corresponding phonological and orthographic representations [40]. But no known studies were committed to investigating whether or not the activation of one type of character information could help with the other type of information-oriented processing of the character.

In conjunction with the findings from Experiment 1, it is intriguing that the *“Dogs’ Catching Mice” effect* occurred both at the lexical and sub-lexical level. One possible account may be that a Chinese character’s interconnected phonological, orthographic, and semantic representations at the lexical level could be easily mapped onto their phonetic-radical counterparts at the sub-lexical level. By way of illustration, the manipulation of the target character in Experiments 1 and 3 pertained to the same character component. That is, the same independent character in Experiment 3 served as the sub-lexical phonetic radical in Experiment 1 as it was combined with another radical to form a phonogram. In this case, the inherent triadic interconnected representations incorporated by the independent character may well be copied over to its sub-lexical variant just as a matter of course.

In short, the results obtained from Experiment 3 did demonstrate that the semantically activated phonetic radicals, when functioning as independent characters, could truly contribute to their phonological recognition. This phenomenon aligns with the *“Dogs’ Catching Mice” effect*, albeit observed at the lexical level.

## 5. General discussion

In Chinese character processing studies, it is tacitly understood that phonetic radicals can contribute to the phonological recognition [18,21,22], and semantic radicals to the semantic recognition of their host phonograms [29–31]. Each abide by the division of labor, without one side’s poking his nose into the other side’s affairs. There seems to exist a “Cats’ Catching Mice” principle governing therein. However, the documentation that phonetic radicals embedded in phonogram characters can be semantically activated provokes us to reason that phonetic radicals, once semantically activated, may need to find an outlet for their sub-lexical semantic representation momentum. It is assumed that the semantically-activated phonetic radicals may influence to their sub-lexical level phonological representation, which has natural affinity and thus regular interface with the host phonogram’s character-level phonological representation. In this manner, phonetic radicals may have blazed stealthily a new path whereby to engage in the host phonogram’s lexical-level phonological retrieval or processing, and thus gains an opportunity to exert an impact, usually beneficial, upon the entire processing event. This new path suggests a great departure from the conventional tacitly-understood path which links the function of phonetic radicals to the function of their host character’s phonology and makes possible phonetic radicals’ direct contribution to their host characters’ processing. This assumption is termed the *“Dogs’ Catching Mice” Conjecture*.

By way of three experiments, the above conjecture was meticulously examined. Experiment 1 was designed to pin down the *“Dogs’ Catching Mice”* phenomena. Experiments 2 and 3 were dedicated to testifying to the charted machinery or path that presumably would give rise to the “Dogs’ Catching Mice” phenomena. The results of Experiment 1 confirmed the seemingly peculiar existence of the *“Dogs’ Catching Mice” effects* by demonstrating that during Chinese phonograms’ phonological recognition process, the semantic activation of phonetic radicals did help to facilitate the character-level phonological processing. The results of Experiment 2 provided evidence for the semantic activation of phonetic radicals during the host phonograms’ recognition, even when their sub-lexical semantic activation would cause interference with that of their host phonograms at the lexical level. The results of Experiment 3, as expectedly, showed that phonetic radicals, when utilized as independent characters, could contribute semantically to their phonological recognition as well, adding credibility to the belief that a character’s lexical-level triadic interconnections of phonology, orthography and semantics might readily transfer to their sub-lexical counterparts and endow them with the corresponding behavioral properties. Put simply, the phonetic radical’s *“Dogs’ Catching Mice”* behavior should be convincingly traced to their independent character counterpart’s *“Dogs’ Catching Mice”* behavior. An embedded phonetic radical, with finesse, is capable of semantically contributing to its host phonogram’s phonological recognition just because its independent counterpart possesses the capability of semantically contributing to its own phonological recognition. This pattern of phonetic radicals, resembling characters, aligns with earlier demonstration that sub-lexical radicals indeed share similar representations with independent characters [32], and independent phonetic radicals in Chinese phonogram phonological recognition undergo lexical processing just like characters [1]. This discovery also further underscores the similarities between radicals and the embedding characters in Chinese character phonological recognition, which reinforces the thought that the difference between radicals and whole characters is ambiguous [1,2]. It needs to be noted, however, that all the phonetic radicals recruited in the present study are free-standing radicals, which in other cases all function as independent characters. It remains indeterminate whether bound phonetic radicals—those that would never stand as independent characters—could behave similarly or not. Studies in this respect would be perspicuously needed in the future.

The moderation effects of phonetic regularity on the phonological processing of the host phonograms, as observed in Experiment 1, also lent support to the presence of the machinery or unconventional path postulated to account for the seeming oddity of a phonetic radical’s contributing semantically, instead of phonologically, to its phonogram’s recognition. The *“Dogs’ Catching Mice”* faciliatory effects were found only for regular and semi-regular phonograms, but not for irregular phonograms, and the regular type displayed a larger size of facilitatory effects than the semi-regular type. The blunt explanation is that regular phonograms, which have identical phonological information to share with their embedded phonetic radicals, have available a highly valid path along which to pass their phonetic radicals’ sub-lexical semantic and phonological activation on to their host characters’ phonology. Semi-regular phonograms, by contrast, only have partial phonological information to share with their phonetic radicals, and thus have available only a partial valid path for that purpose. As for irregular phonograms, which by definition, were devoid of any phonological commonality with their embedded phonetic radicals, there might exist no such path at all.

Taken together, the three experiments provided clear evidence for the existence of the *“Dogs’ Catching Mice” Path.* It may be true that in character processing, phonetic radicals may engage themselves with their host phonograms’ phonological processing by tracing two paths. One is to act upon the host phonogram’s phonology by phonologically activating themselves. The other is to act upon the host phonogram’s phonology by first semantically activating and then phonologically activating themselves. The former stands for the more commonly-accepted, direct *“Cats’ Catching Mice” Path,* while the latter for the more novel, indirect *“Dogs’ Catching Mice” Path.* The *“Dogs’ Catching Mice” Conjecture* seemed to gain support.

This dual path not only enriches our theoretical understanding of Chinese phonogram processing but also offers practical insights for language education and computational applications. First, it expands our understanding of the functional roles of phonetic radicals, revealing that their contributions are more dynamic and multifaceted than previously thought. The discovery of the *“Dogs’ Catching Mice” Path* underscores the interconnectedness of semantic and phonological processing at both sub-lexical and lexical levels, providing new insights into the representational and processing mechanisms of Chinese phonograms. Second, the findings have significant implications for Chinese language education and computational models of character processing. For educators, understanding the dual roles of phonetic radicals can inform teaching strategies that leverage both phonological and semantic cues to enhance character recognition and reading fluency. For computational linguists, this dual-pathway model can improve the accuracy of natural language processing systems by incorporating the semantic contributions of phonetic radicals.

One limitation of the current study, as noted by a reviewer, is the potential influence of collocation associations between primes and targets in Experiment 3. For instance, some prime-target pairs (e.g., “时” and “表”) may form commonly used phrases (e.g., “时间表”), which could introduce confounding effects. While we carefully controlled for phonological and semantic relationships, completely eliminating collocational biases proved challenging due to the inherent structure of the Chinese language and the need to maintain experimental validity. Future studies could address this issue by employing stricter controls or alternative paradigms to further minimize such influences.

## 6. Conclusion

In character processing studies, it is an old story that phonetic radicals can act upon and thus contribute to their host phonograms’ phonological processing by phonologically activating themselves at the sub-lexical level. The present study, however, related a much new story that phonetic radicals can act upon and thus contribute to their host phonograms’ phonological processing by way of semantically, in contrast to phonologically, activating themselves at the sub-lexical level. The new story is labelled *the “Dogs’ Catching Mice” Conjecture*. By employing two primed phonological readability tasks (Experiments 1 and 3) and one primed part-of-speech judgement task (Experiment 2), endorsing evidences were proffered for this speculation.

The significance of the present study lies in the conjecture that, in Chinese-speakers’ daily recognition of phonogram characters, it may be a matter of fact that phonetic radicals have forged two paths in contributing to their phonograms’ phonological processing. The commonly-accepted, more direct *“Cats’ Catching Mice” Path* may always go hand in hand with the largely-ignored somewhat indirect *“Dogs’ Catching Mice” Path*. The two distinct paths may interact in a manner far exceeding our current understanding. More diverse and converging evidences in this respect are undoubtedly called for in the future. It is now desirable to claim that researchers allocate more efforts to the yet-to-be more finely-charted new path. One of the imperatives for the next study is to put the non-free standing phonetic radicals in the spotlight.

## Supporting information

S1 Table Primes and Targets Used in Experiments 1 and 2. (DOCX)

S2 Table Primes and Targets Used in Experiment 3. (DOCX)
