# Supplementary material for: The “Dogs’ Catching Mice” conjecture in Chinese phonogram processing
Source: PLoS One. 2025 Jun 6;20(6):e0324848. doi: 10.1371/journal.pone.0324848 (PMC12143521; doi:10.1371/journal.pone.0324848)
Supplement: S1 Table — (DOCX) [file pone.0324848.s001.docx]

| **Item No.** | **Semantically Related (SR) Primes** | **Semantically Unrelated (SU) Primes** | **Targets** | | **Phonetic Radicals of the Targets** |
| --- | --- | --- | --- | --- | --- |
| **1** | 时 | 柜 | 裱/biao3/ | Regular Phonograms | 表/biao3/ |
| **2** | 晕 | 蛮 | 婚/hun1/ |  | 昏/hun1/ |
| **3** | 尾 | 身 | 沫/mo4/ |  | 末/mo4/ |
| **4** | 美 | 窄 | 锈/xiu4/ |  | 秀/xiu4/ |
| **5** | 危 | 痒 | 鞍/an1/ |  | 安/an1/ |
| **6** | 狼 | 神 | 洋/yang2/ |  | 羊/yang2/ |
| **7** | 婶 | 帐 | 淑/shu1/ |  | 叔/shu1/ |
| **8** | 绿 | 胖 | 蜻/qing1/ |  | 青/qing1/ |
| **9** | 内 | 正 | 鲤/li3/ |  | 里/li3/ |
| **10** | 客 | 宝 | 缤/bin1/ |  | 宾/bin1/ |
| **11** | 全 | 蜜 | 谐/xie2/ | Semi-regular Phonograms | 皆/jie1/ |
| **12** | 爬 | 选 | 橙/cheng2/ |  | 登/deng1/ |
| **13** | 傲 | 嫣 | 逛/guang4/ |  | 狂/kuang2/ |
| **14** | 独 | 祥 | 婵/chan2/ |  | 单/dan1/ |
| **15** | 说 | 跳 | 唁/yan4/ |  | 言/yan2/ |
| **16** | 杀 | 参 | 惭/can2/ |  | 斩/zhan3/ |
| **17** | 低 | 娇 | 犒/kao4/ |  | 高/gao1/ |
| **18** | 妖 | 钱 | 愧/kui4/ |  | 鬼/gui3/ |
| **19** | 圆 | 困 | 访/fang3/ |  | 方/fang1/ |
| **20** | 友 | 夫 | 绷/beng1/ |  | 朋/peng2/ |
| **21** | 走 | 夸 | 涉/she4/ | Irregular Phonograms | 步/bu4/ |
| **22** | 脚 | 辫 | 捉/zhuo1/ |  | 足/zu2/ |
| **23** | 笑 | 吊 | 烁/shuo4/ |  | 乐/le4/ |
| **24** | 想 | 拿 | 腮/sai1/ |  | 思/si1/ |
| **25** | 愉 | 胖 | 掀/xian1/ |  | 欣/xin1/ |
| **26** | 齿 | 头 | 邪/xie2/ |  | 牙/ya2/ |
| **27** | 果 | 桌 | 弧/hu2/ |  | 瓜/gua1/ |
| **28** | 剑 | 词 | 切/qie1/ |  | 刀/dao1/ |
| **29** | 关 | 出 | 妍/yan2/ |  | 开/kai1/ |
| **30** | 入 | 斗 | 拙/zhuo2/ |  | 出/chu1/ |

**S1 Table. Primes and Targets Used in Experiments 1 and 2**
